# Supplementary material for: Rapid human oogonia-like cell specification via transcription factor-directed differentiation
Source: EMBO Rep. 2025 Jan 23;26(4):1114–43. doi: 10.1038/s44319-025-00371-2 (PMC11850904; doi:10.1038/s44319-025-00371-2)
Supplement: Supplementary file 1 — Appendix [file 44319_2025_371_MOESM1_ESM.pdf]

Appendix

Rapid Human Oogonia-like Cell Specification via Transcription Factor-Directed Differentiation

Merrick Pierson Smela,[1,2] Christian C Kramme,[1,2,†] Patrick R.J. Fortuna,[1,2] Bennett Wolf,[1,2] Venkata Srikar Kavirayuni,[3] Jessica Adams,[1,2] Carl Ma,[1,2] Sergiy Velychko,[1,2,] Ursula Widocki,[4] Shrey Goel,[3] Tianlai Chen,[3] Sophia Vincoff,[3] Edward Dong,[1,2] Richie E. Kohman,[1,2] Mutsumi Kobayashi,[5] Toshi Shioda,[6] George M. Church,[1,2] Pranam Chatterjee[3,7,8]

1. Wyss Institute, Harvard Medical School
2. Department of Genetics, Harvard Medical School
3. Department of Biomedical Engineering, Duke University
4. Broad Institute of Harvard and MIT
5. Department of Obstetrics and Gynaecology, School of Medicine, Juntendo University
6. Massachusetts General Hospital Center for Cancer Research, Harvard Medical School
7. Department of Computer Science, Duke University
8. Department of Biostatistics and Bioinformatics, Duke University

[†]Corresponding author: christian@gametogen.com

Appendix Table of Contents

1. Appendix Figure S1. Expression of prioritized transcription factors in the human fetal gonad atlas……..3
2. Appendix Figure S2. Development of dual fluorescent reporter hiPSC line for TF screening……………4
3. Appendix Figure S3. Development of doxycycline inducible TF screening cell lines………………………6
4. Appendix Figure S4. CellTypist and UMAP annotation of transcriptomic data based on cell types in the human fetal gonad reference atlas…………………………………………………………………………7
5. Appendix Figure S5: Differential gene expression analysis and GO term analysis of iOLC induction methods……………………………………………………………………………………………………8
6. Appendix Figure S6. Representative flow cytometry analysis………………………………………………..9

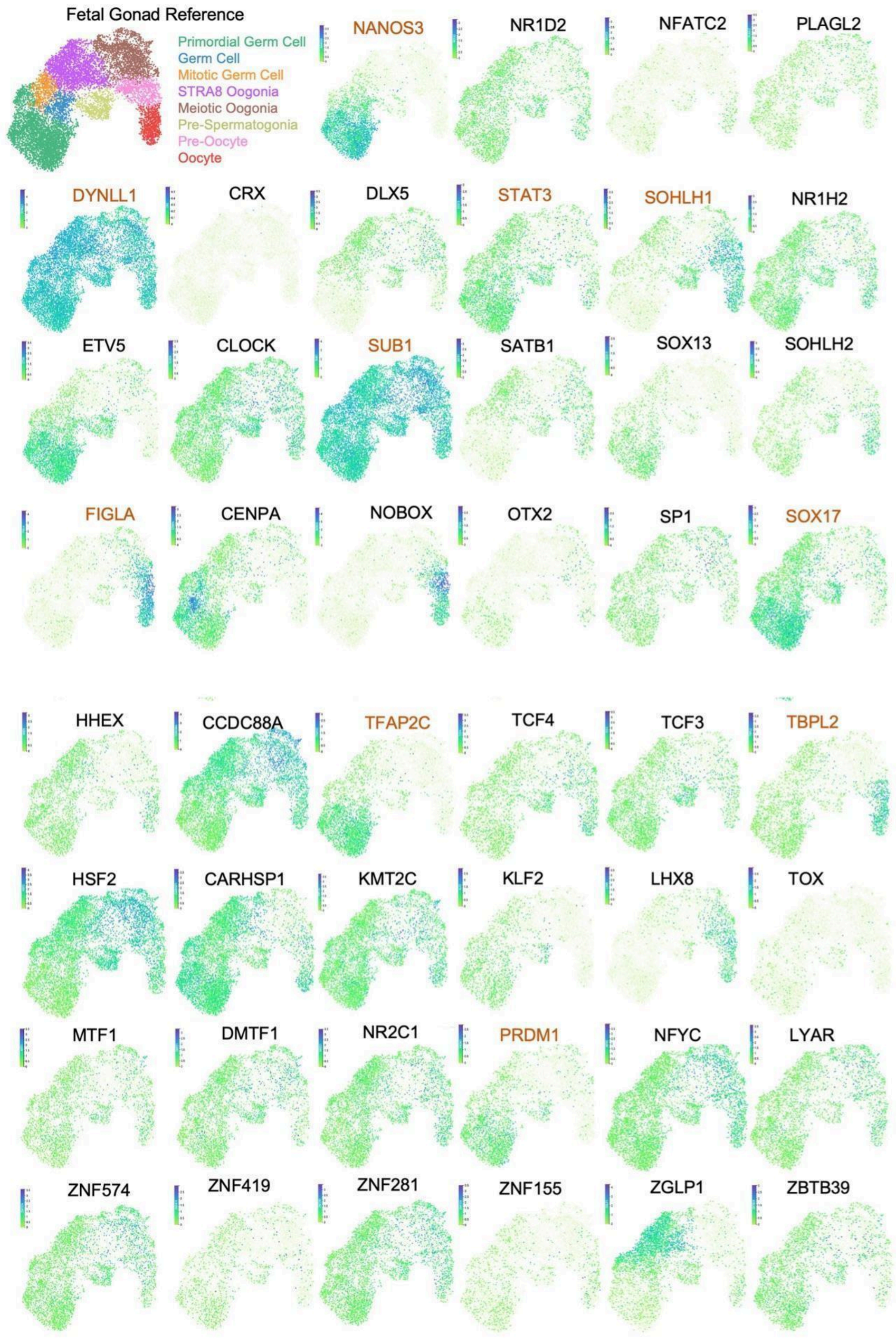

Appendix Figure S1. Expression of prioritized transcription factors in the human fetal gonad atlas**, Related to Figure 1.** The expression of all 46 TFs was visualized using the reproductive cell atlas, utilizing only germline cells obtained from the fetal atlas of Garcia-Alonso et al. 2022. Expression level for each TF is mapped onto the UMAP, with the cell type annotation shown in the reference map. TFs included as controls in the study are highlighted orange.

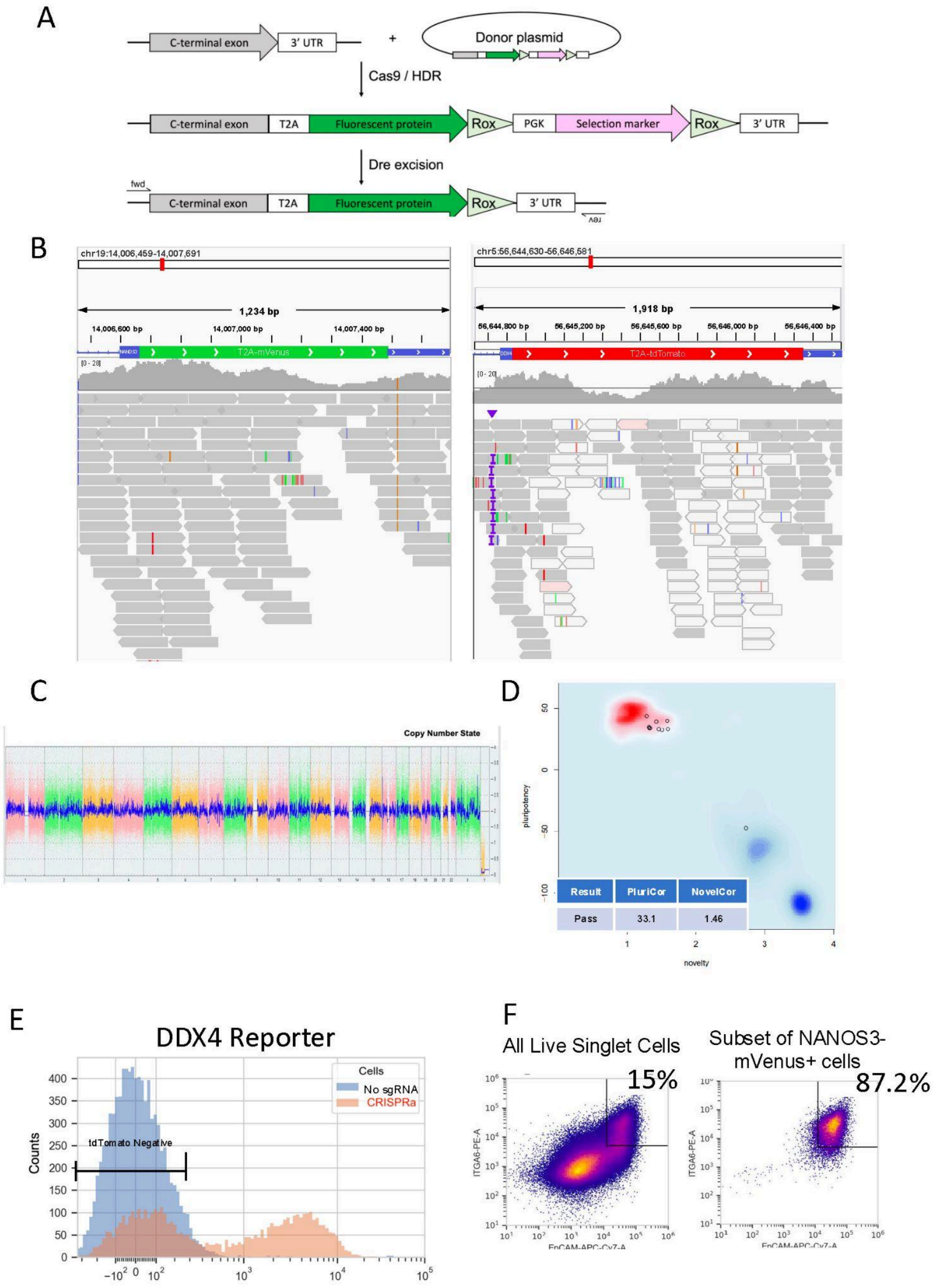

Appendix Figure S2. Development of dual fluorescent reporter hiPSC line for TF screening**, Related to Figure 2.** A) Schematic representation of cell line generation methodology. B) Genotyping WGS of the reporter line utilized in study, with specific display of the inserted reporter construct regions. C) The whole genome view displays all somatic and sex chromosomes in one frame with high level copy number. The smooth signal plot (right y-axis) is the smoothing of the log2 ratios which depict the signal intensities of probes on the microarray. A value of 2 represents a normal copy number state (CN = 2). A value of 3 represents chromosomal gain (CN = 3). A value of 1 represents a chromosomal loss (CN = 1). The pink, green and yellow colors indicate the raw signal for each individual chromosome probe, while the blue signal represents the normalized probe signal

which is used to identify copy number and aberrations D) The pluripotency plot window provides a visual representation of the tested samples in the analysis. The pluripotency and novelty x/y scatter plot combines the pluripotency score on the y-axis with the novelty score on the x-axis. The red and blue background hint to the empirical distribution of the pluripotent (red) and non-pluripotent (blue) samples in the reference data set. The samples were analyzed using an algorithm that integrates gene expression data to authenticate pluripotency status. Samples are screened against samples in the stem cell database and given a pluripotency score (PluriCor) and novelty score (NovelCor), which are shown in the table. Pass shows a clear pluripotency signature. Fail means the samples are not pluripotent. A non-iPSC sample was used in this experiment to serve as a negative control for non-pluripotency. E) Flow cytometry analysis after induction of the DDX4-reporter locus through CRISPR-activation, blue represents a no sgRNA control and red represents the CRISPRa- sgDDX4 condition, the bracket highlights the cells considered reporter negative. F) Flow cytometry analysis of hPGCLC formation using floating aggregate method (Sasaki et al. 2015) utilized in Figure EV2 for the D4TN3V reporter line. Live, single cells are visualized for expression of the cell surface markers EpCAM and ITGA6 (left panel). Additionally in the subset of NANOS3-mVenus+ cells, expression of EpCAM/ITGA6 is visualized (right panel).

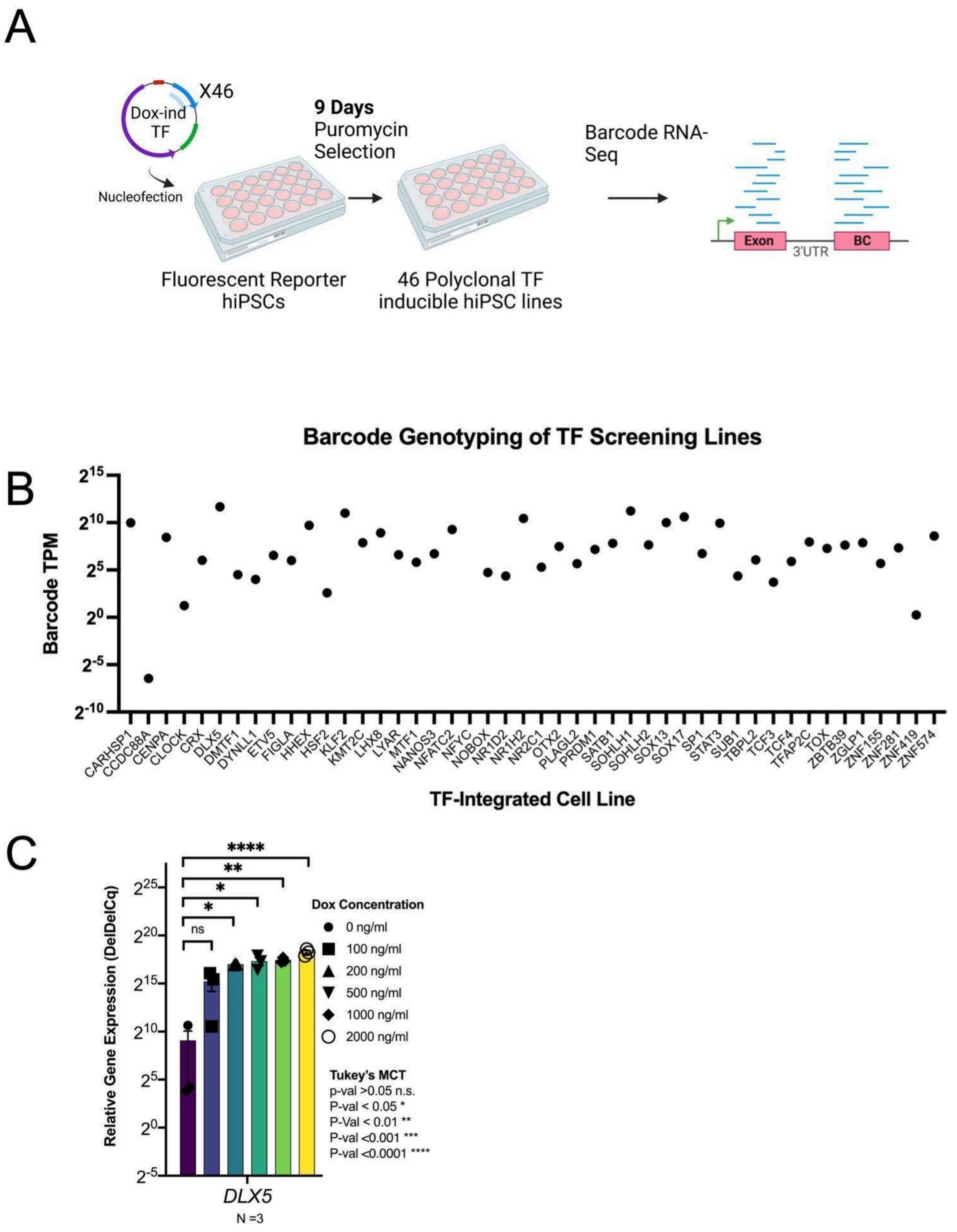

Appendix Figure S3. Development of doxycycline inducible TF screening cell lines**, Related to Figure 2.** Schematic representation of 46 cell line generation B) Genotyping via barcode capture through RNA-seq. Transcriptomes were captured and analyzed via Kallisto for barcode identification. Barcode transcripts per million (TPM) from Kallisto analysis of 46 cell lines. The Barcode TPM for each intended TF is visualized for each line. C) RT-qPCR of the *DLX5* gene under doxycycline serial dilution in three independent replicates plotted relative to *GAPDH* using the ∆∆Cq method. Statistical analysis is performed using ANOVA with multiple comparison testing.

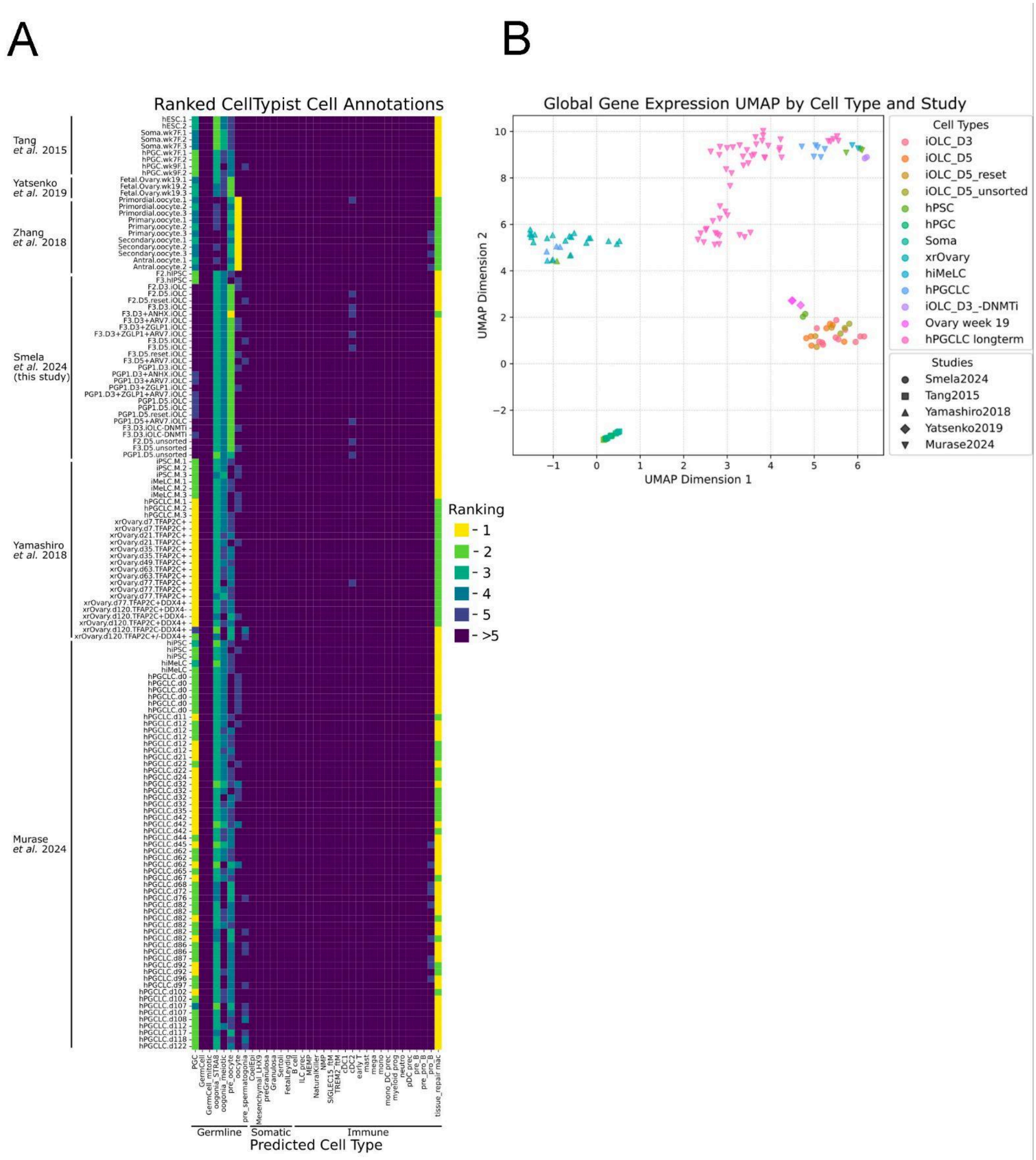

Appendix Figure S4. CellTypist and UMAP annotation of transcriptomic data based on cell types in the human fetal gonad reference atlas. **Related to Figure 5.** A. For each sample, the top five cell types predicted by CellTypist are shown. The cell types are labeled at the bottom, grouped by germline, somatic, and immune lineages. The samples are labeled on the left, grouped by study. B. UMAP clustering of whole transcriptomic data is performed for all samples utilized in (a).

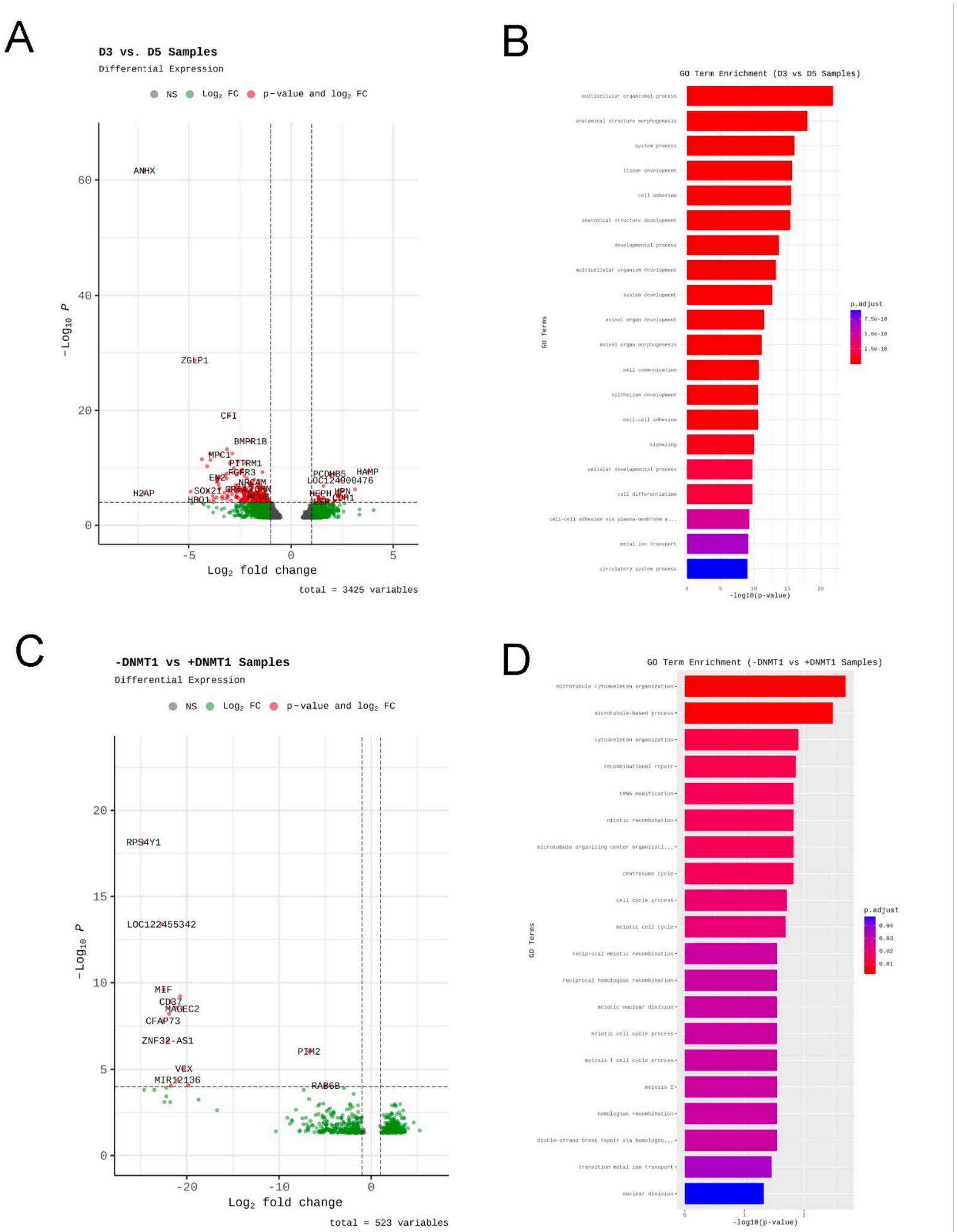

Appendix Figure S5. Differential gene expression analysis and GO term analysis of iOLC induction methods. **Related to Figure 5.** A. Volcano plot of differential gene expression analysis of D3 derived iOLCs versus D5 derived iOLCs, with the x-axis plotting log2FC in expression and y-axis plotting the adjusted p-value. B. Significantly enriched GO terms of significantly differentially expressed genes between D3 and D5-derived iOLCs. C. Volcano plot of differential gene expression analysis of D3 derived iOLCs without DNMTi versus all DNMTi treatment-derived iOLCs, with the x-axis plotting log2FC in expression and y-axis plotting the adjusted p-value. B. Significantly enriched GO terms of significantly differentially expressed genes between D3 derived iOLCs without DNMTi versus all iOLCs derived from DNMTi treatments.

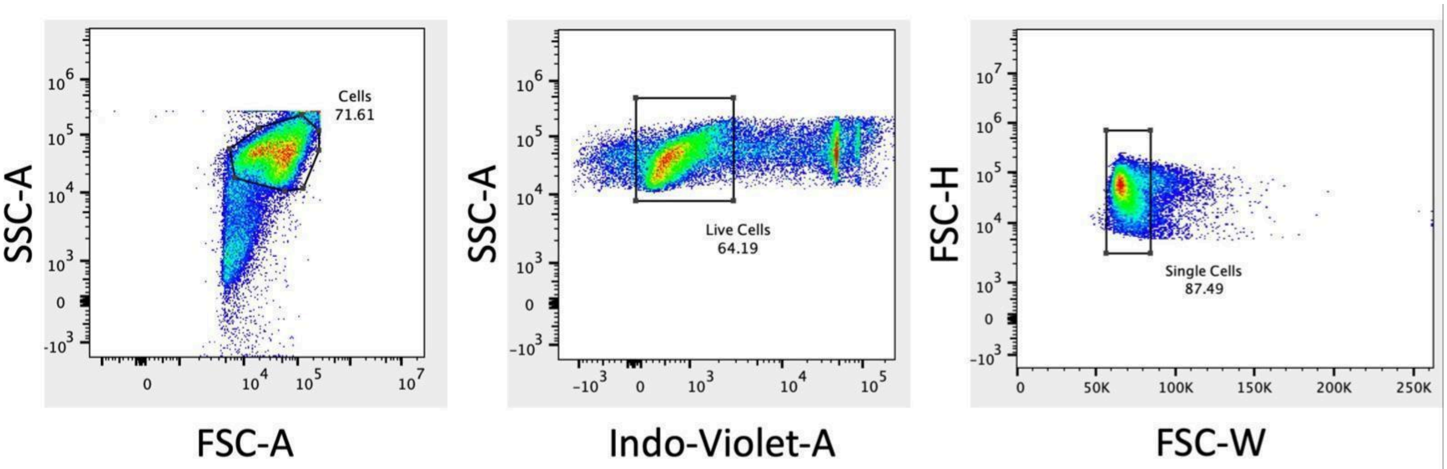

Appendix Figure S6: Representative flow cytometry analysis, **Related to Figure 2, 3** A) Gating strategy for identifying cell events from debris using SSC-A versus FSC-A dispersion (left panel), live cells from dead cells using SSC-A by Indo-Violet-A dispersion in DAPI stained samples (center panel), and live single cells using FSC-H by FSC-W dispersion (right panel). A minimum of 10,000 live singlets were used for analysis. Analysis was performed in Flowjo software.
