# Supplementary figures and images for: Rapid human oogonia-like cell specification via transcription factor-directed differentiation
Source: EMBO Rep. 2025 Jan 23;26(4):1114–43. doi: 10.1038/s44319-025-00371-2 (PMC11850904; doi:10.1038/s44319-025-00371-2)

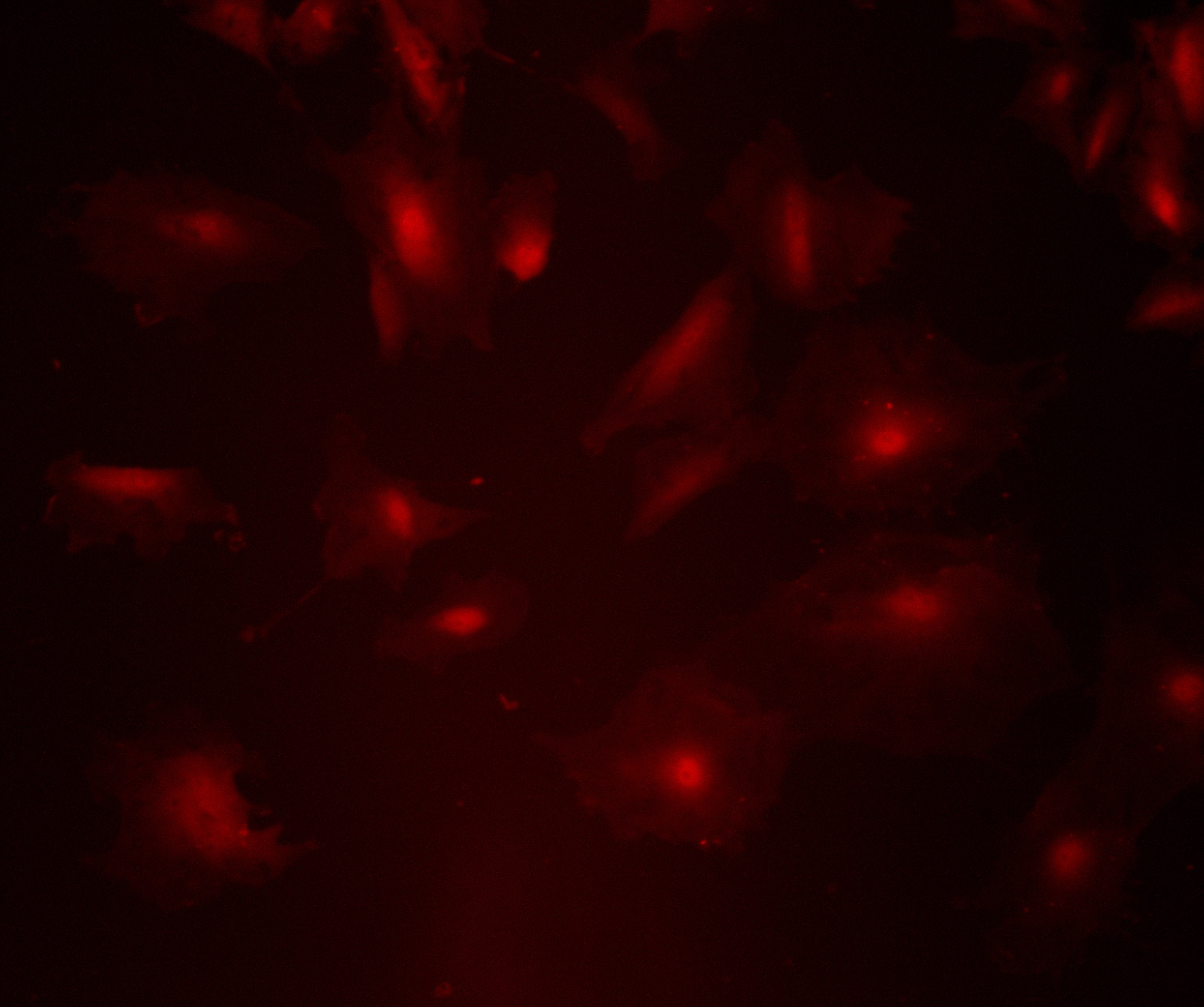

Supplement: Supplementary file 9 — Source data Fig. 4 [file 44319_2025_371_MOESM9_ESM.zip › Figure 4A Raw Data/D3_DDX4_0000_texred-Day 10.jpg]

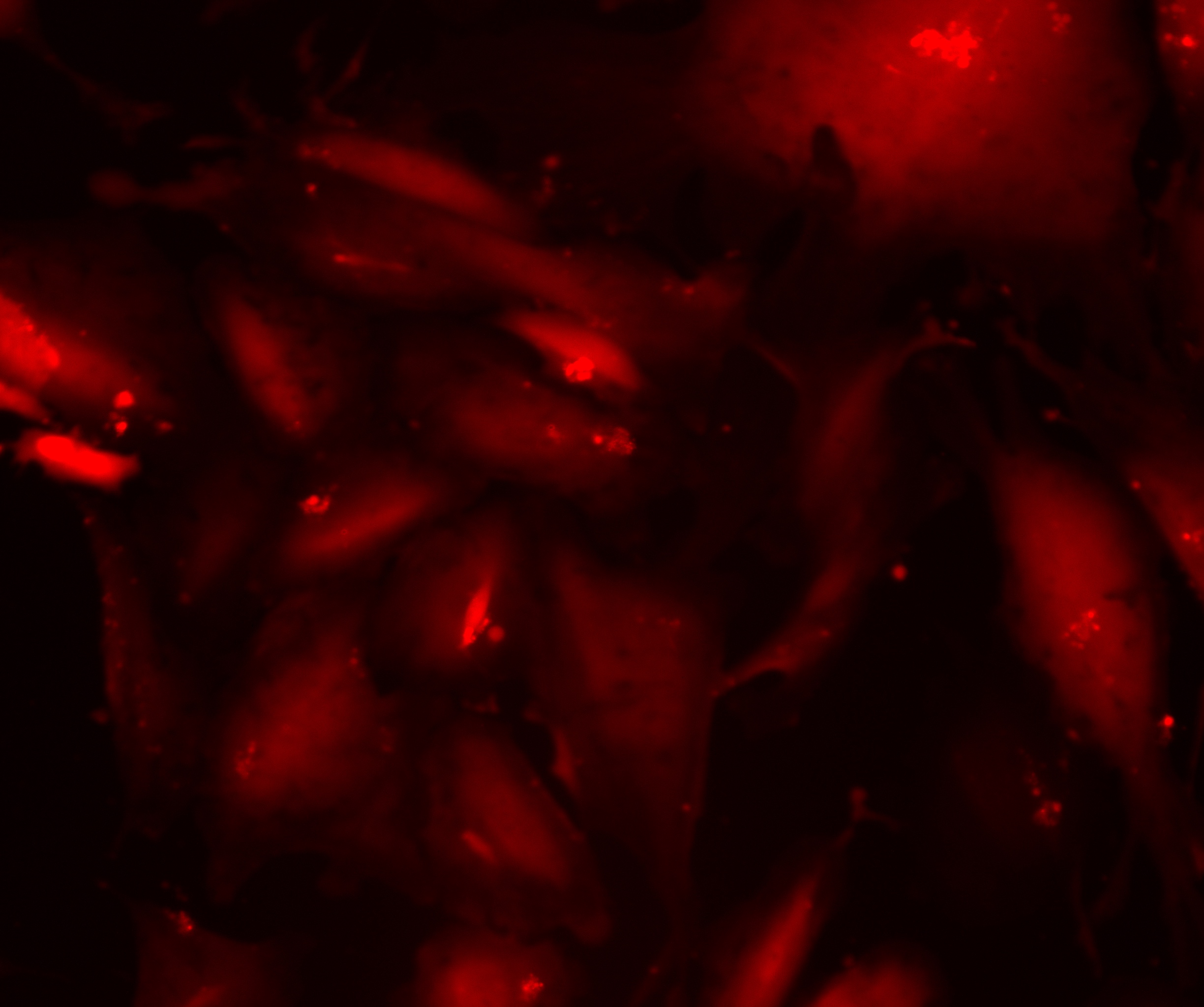

Supplement: Supplementary file 9 — Source data Fig. 4 [file 44319_2025_371_MOESM9_ESM.zip › Figure 4A Raw Data/D3_DDX4_0002_texred-Day 28.jpg]

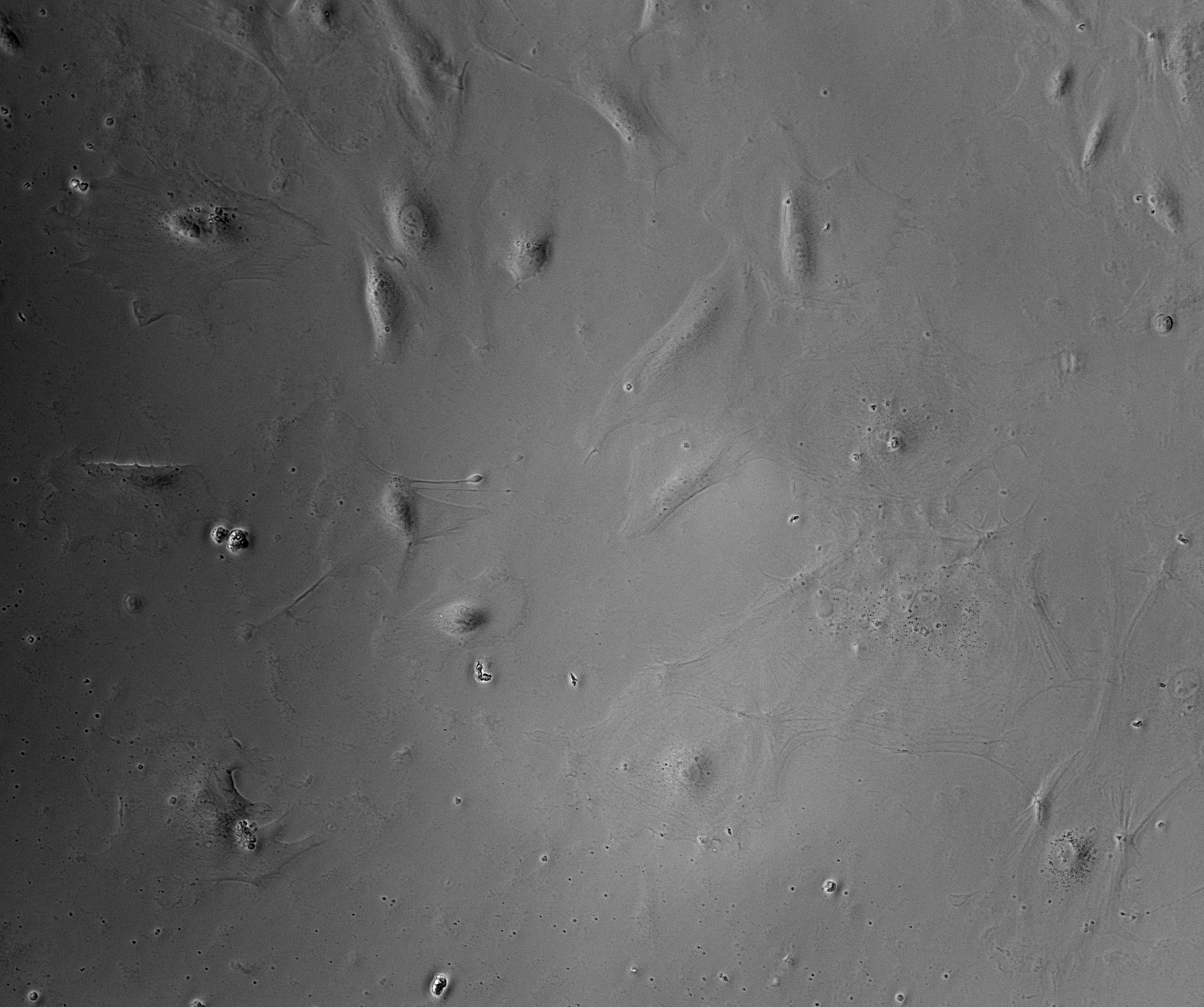

Supplement: Supplementary file 9 — Source data Fig. 4 [file 44319_2025_371_MOESM9_ESM.zip › Figure 4A Raw Data/D3_DDX4_0000_trans-Day 10.jpg]

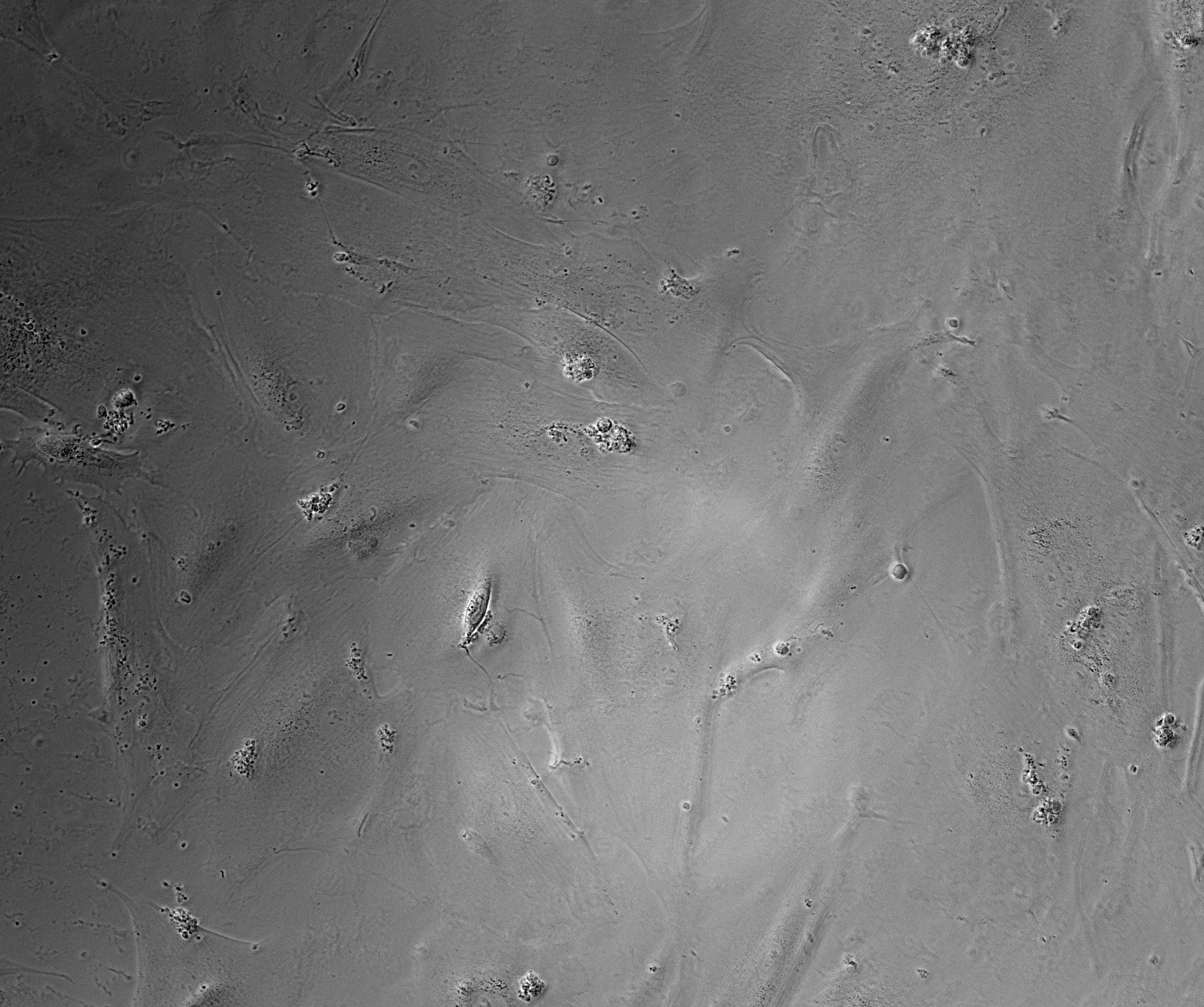

Supplement: Supplementary file 9 — Source data Fig. 4 [file 44319_2025_371_MOESM9_ESM.zip › Figure 4A Raw Data/D3_DDX4_0002_trans-Day 28.jpg]
