# Supplementary material for: Rapid human oogonia-like cell specification via transcription factor-directed differentiation
Source: EMBO Rep. 2025 Jan 23;26(4):1114–43. doi: 10.1038/s44319-025-00371-2 (PMC11850904; doi:10.1038/s44319-025-00371-2)
Supplement: Supplementary file 10 — Expanded View Figures [file 44319_2025_371_MOESM10_ESM.pdf]

Expanded View Figures

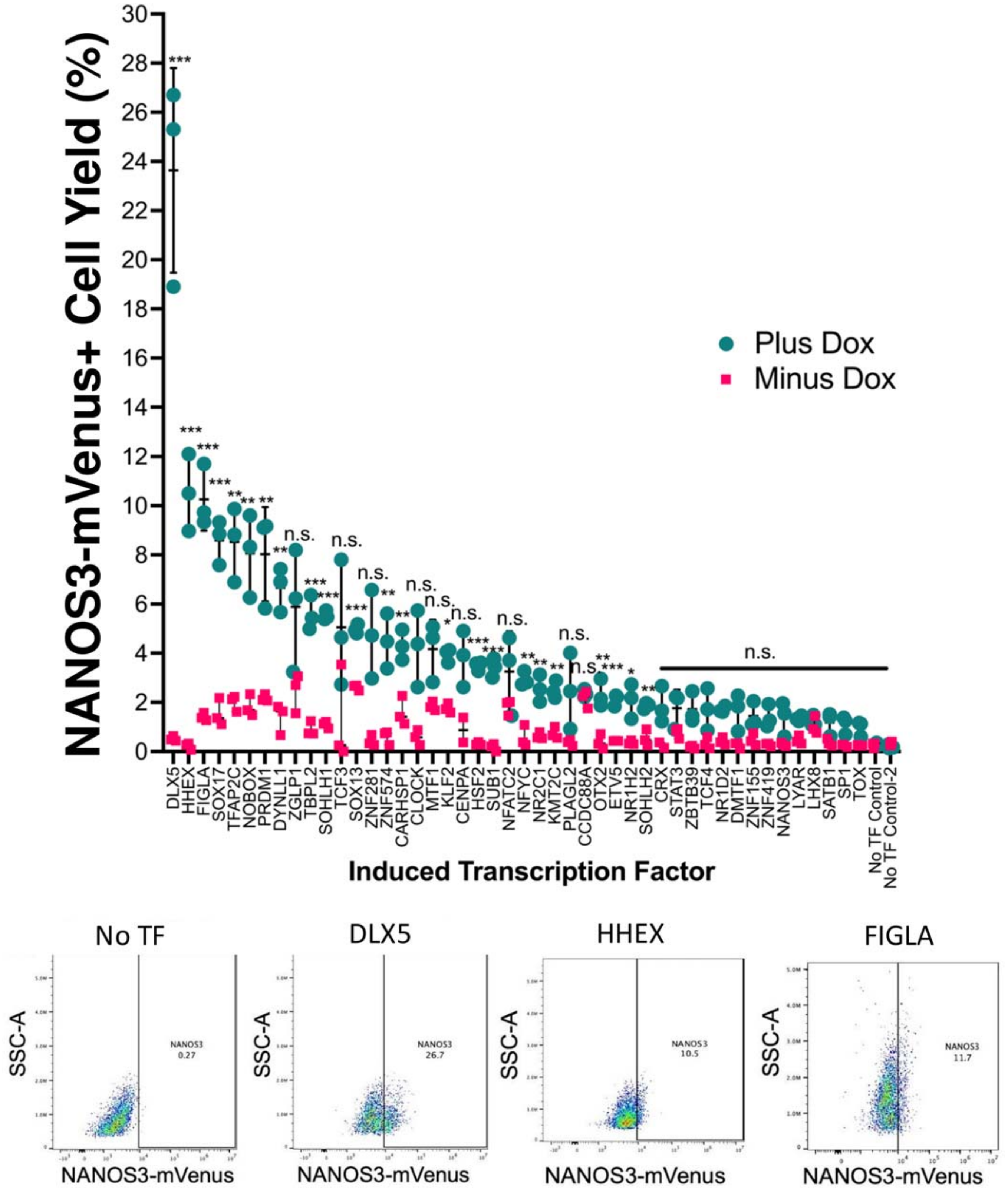

Figure EV1. Single TF-overexpression screening for NANOS3+ cell yield, Related to Fig. 2.

Results for NANOS3-T2A-mVenus flow cytometry for triplicate induction conditions in the plus (blue) and minus (pink) doxycycline induction condition for each TF. Data were plotted as a percent of NANOS3-T2A-mVenus+ cells on the y-axis for each TF. Individual dots represent individual wells of the induction condition, seeded separately from a single hiPSC line. The horizontal black line represents the mean of induction replicates. Statistical significance was determined by multiple *T*-test comparison between the plus and minus dox condition for each TF, with a *p* value <0.05 considered as significant. The FDR correction was utilized for multiple hypothesis testing. ***$p < 0.001$, **$P < -0.01$, *$p < 0.05$. Representative flow cytometry plots are shown for the No-TF control condition and the three highest yielding conditions: DLX5, HHEX, and FIGLA.

 © The Author(s)

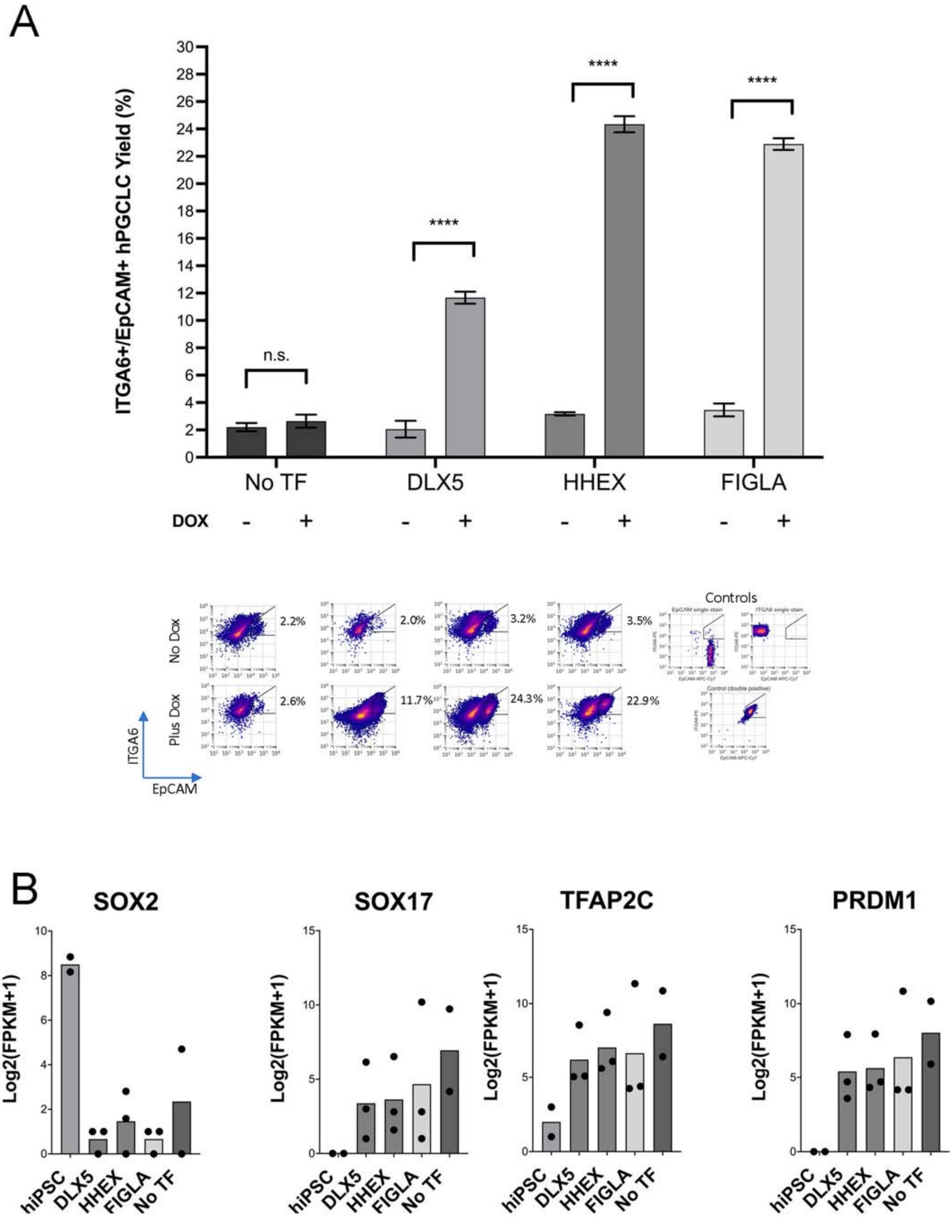
A
ITGA6+/EpCAM+ hPGCLC Yield (%)
n.s.
****
****
****
No TF
DLX5
HHEX
FIGLA
DOX - + - + - + - +
No Dox
Plus Dox
2.2%
2.0%
3.2%
3.5%
2.6%
11.7%
24.3%
22.9%
Controls
ITGA6
EpCAM
B
SOX2
SOX17
TFAP2C
PRDM1
Log2(FPKM+1)
hiPSC
DLX5
HHEX
FIGLA
No TF

Figure EV2. *DLX5*, *HHEX*, and *FIGLA* overexpression significantly improves hPGCLC yield, Related to Fig. 2.

(**A**) Generation of hPGCLCs in $N = 3$ replicates of $n = 9$ pooled embryoid bodies per condition. hPGCLCs were induced according to the protocol from Sasaki et al, 2015 and analyzed via flow cytometry for the markers EpCAM and ITGA6. hPGCLCs were induced in the presence and absence of doxycycline in lines harboring inducible vectors for *DLX5*, *HHEX*, and *FIGLA* and a no TF control. Statistical analysis was performed via an unpaired two-tailed *t*-test between doxycycline and no doxycycline conditions for each condition. ****$p < 0.0001$. Representative flow cytometry graphs are shown for EpCAM/ITGA6 on live singlets for the no dox and plus dox conditions as well as single stain controls. (**B**) Expression of known hPGCLC marker genes (*SOX17*, *TFAP2C*, *PRDM1*) and hiPSC marker gene (*SOX2*), in NANOS3+ sorted cells isolated from monolayer induction in the no TF control, *DLX5*, *HHEX*, and *FIGLA* overexpression conditions. RNA abundance was measured via RNA-seq for $N = 2$ or 3 independent replicates and plotted via Log2 normalization of FPKM + 1 values against an hiPSC control.

 © The Author(s)

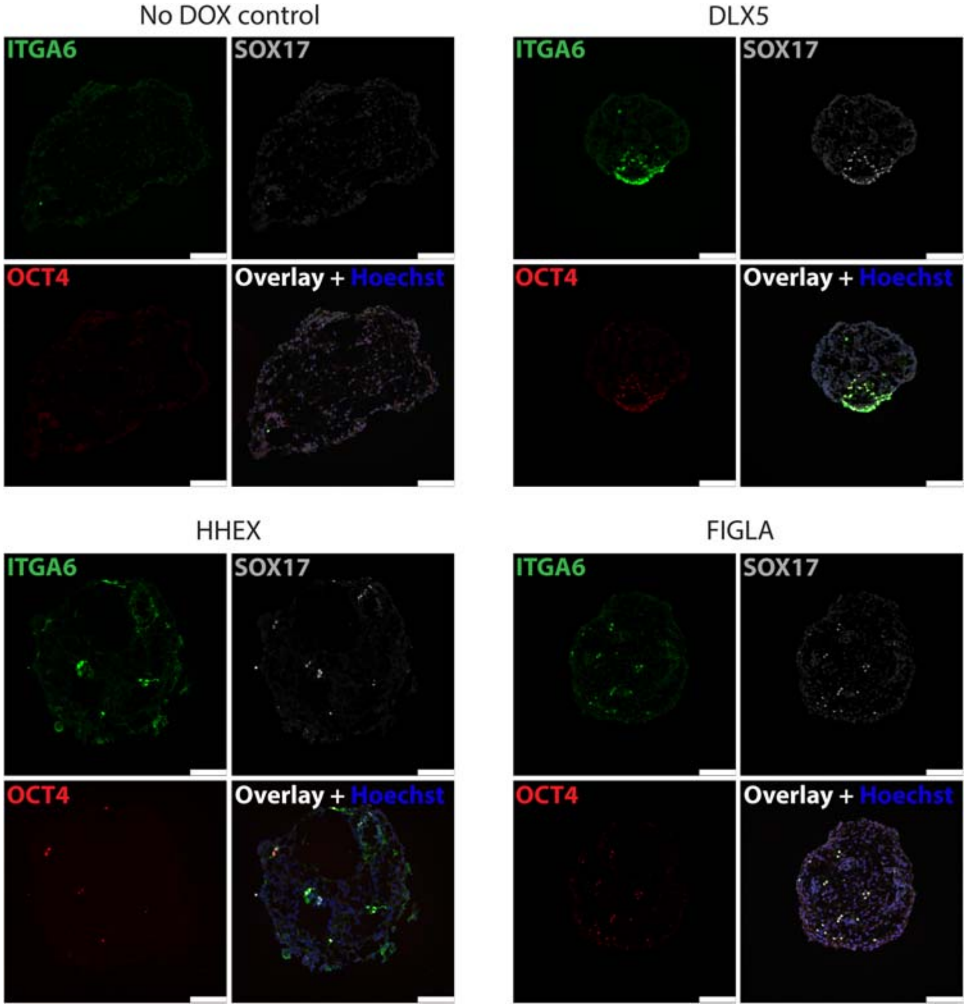

Figure EV3. TF-derived hPGCLCs display canonical protein expression in floating aggregate differentiation, Related to Fig. 2.

hPGCLCs were induced according to the protocol from Sasaki et al, 2015 and analyzed via immunofluorescence after fixation and cryosectioning. hPGCLC markers ITGA6 (green), SOX17 (gray), and OCT4 (red) are visualized alongside Hoechst staining (blue). A no doxycycline control and embryoid bodies from *DLX5*, *HHEX*, and *FIGLA* inductions are shown at day 4 of differentiation. Scale bars are 100 µm.

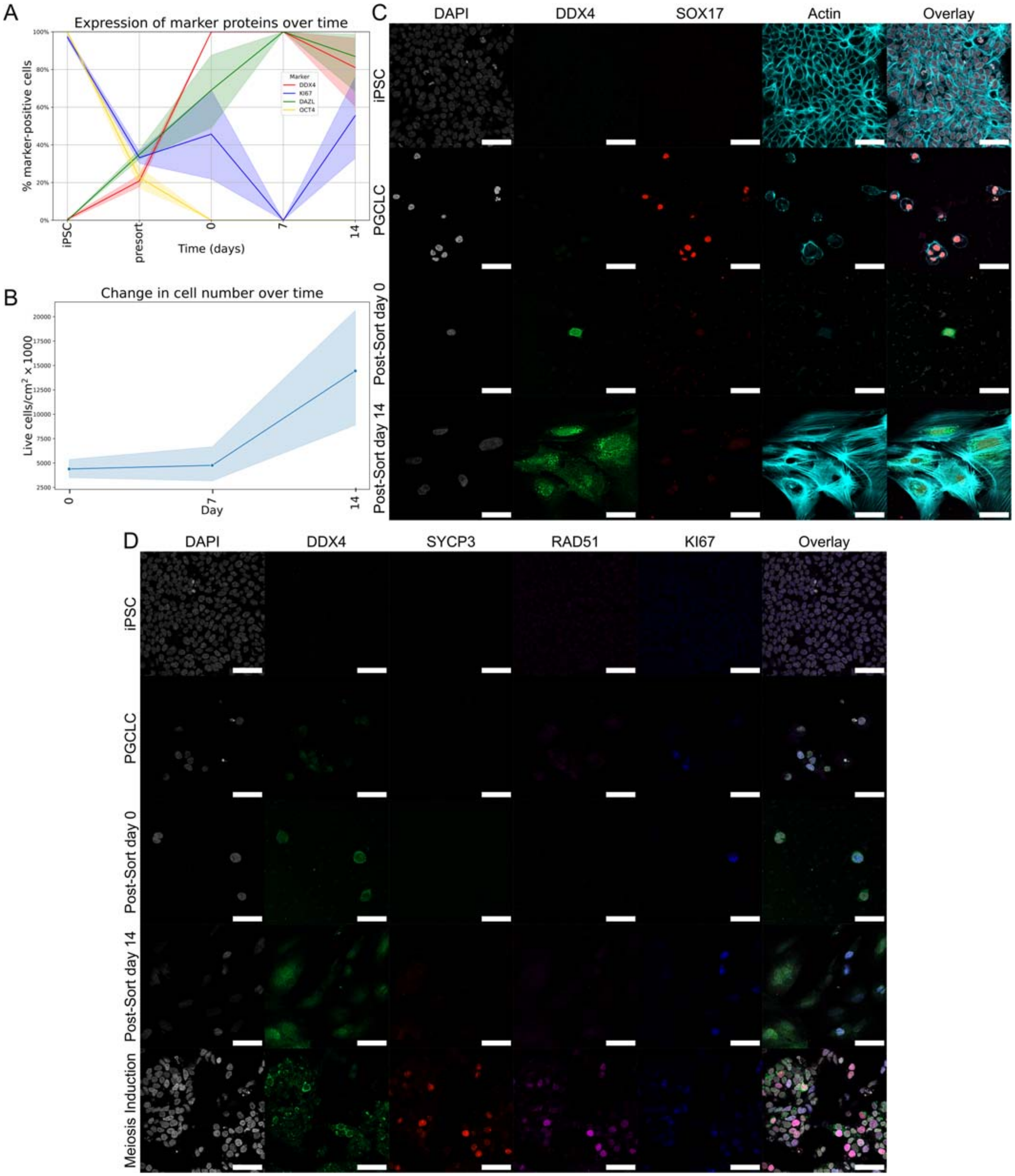
A
Expression of marker proteins over time
% marker-positive cells
Time (days)
iPSC
presort
0
7
14
Marker
DDX4
KI67
DAZL
OCT4
B
Change in cell number over time
Live cells/cm² × 1000
Day
C
DAPI
DDX4
SOX17
Actin
Overlay
iPSC
PGCLC
Post-Sort day 0
Post-Sort day 14
D
DAPI
DDX4
SYCP3
RAD51
KI67
Overlay
iPSC
PGCLC
Post-Sort day 0
Post-Sort day 14
Meiosis Induction

 © The Author(s)

Figure EV4. Staining of iOLCs for PGC and meiosis marker proteins. Related to Fig. 3.

(**A**) Percentage of marker-positive cells at different timepoints of iOLC induction and culture post-sorting. $N = 3$ biological replicates (F2, F3, PGP1) with at least two images per cell line. (**B**) The total number of cells over culture post-sorting. (**C**) Staining of hiPSCs, hPGCLCs, and sorted iOLCs (day 0 and day 14 post-sort) for DDX4 (green), SOX17 (red), and actin (cyan). Scale bars are 50 µm. (**D**) Staining for DDX4 (green), SYCP3 (red), RAD51 (magenta), and KI67 (blue). As a positive control for meiotic markers, staining was performed on cells differentiated using the meiosis induction protocol described in Pierson Smela et al 2024. Scale bars are 50 µm.

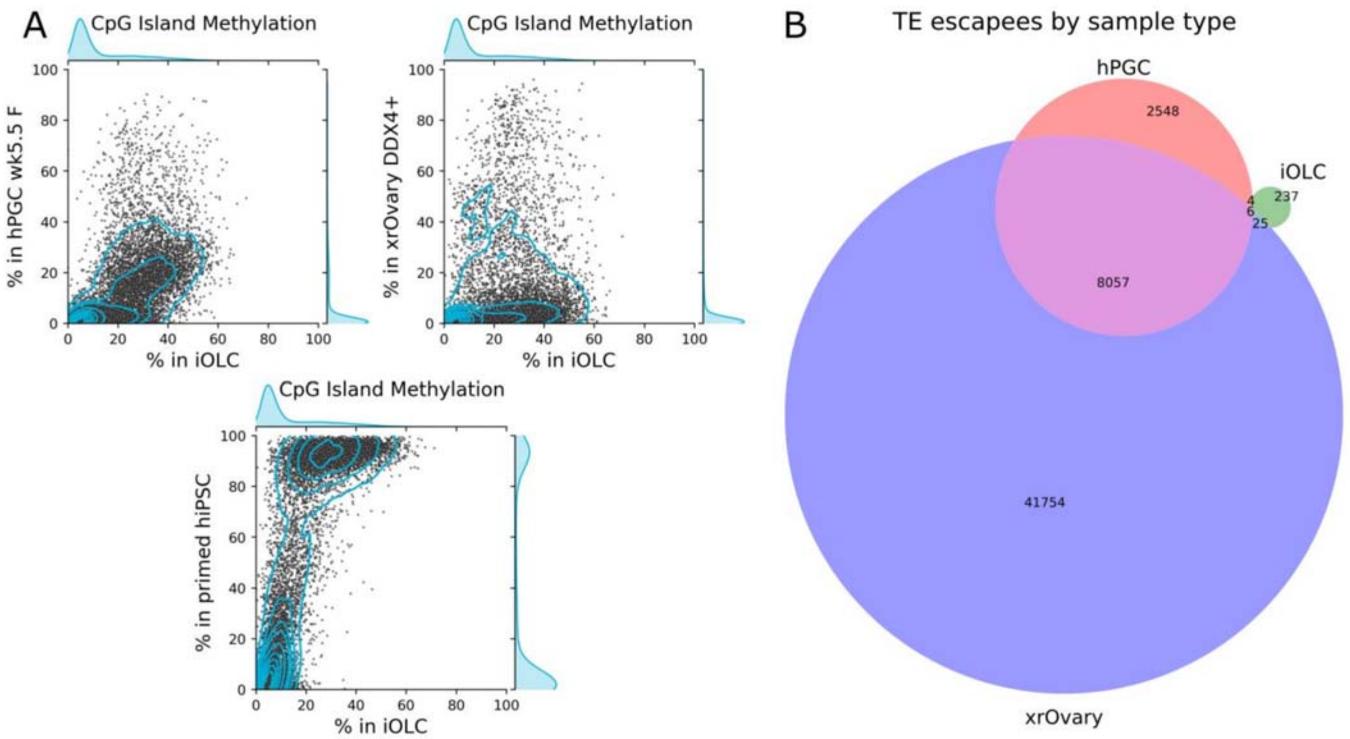

Figure EV5. Additional DNA methylation analysis of iOLCs, hPGCs, and xrOvary cells. Related to Fig. 6.

(**A**) CpG island methylation in primed hiPSCs, iOLCs (induced using D5 TFs and the improved protocol), week 5.5 female hPGCs, and xrOvary day 120 DDX4+ cells. (**B**) Overlap of TEs escaping demethylation (defined as >80% average methylation) in iOLCs, week 5.5 female hPGCs, and xrOvary day 120 DDX4+ cells.

 © The Author(s)
